# Rapid evolution of *avirulence* genes in rice blast fungus *Magnaporthe oryzae*

**DOI:** 10.1186/1471-2156-15-45

**Published:** 2014-04-11

**Authors:** Ju Huang, Weina Si, Qiming Deng, Ping Li, Sihai Yang

**Affiliations:** 1 State Key Laboratory of Pharmaceutical Biotechnology, School of Life Sciences, Nanjing University, Nanjing 210093, China; 2 Rice Research Institute, Sichuan Agricultural University, Wenjiang 611130, China

**Keywords:** *Magnaporthe oryzae*, *Avr*-genes, Evolutionary features, Rapid evolution

## Abstract

**Background:**

Rice blast fungus *Magnaporthe oryzae* is one of the most devastating pathogens in rice. Avirulence genes in this fungus share a gene-for-gene relationship with the resistance genes in its host rice. Although numerous studies have shown that rice blast *R*-genes are extremely diverse and evolve rapidly in their host populations, little is known about the evolutionary patterns of the *Avr*-genes in the pathogens.

**Results:**

Here, six well-characterized *Avr*-genes and seven randomly selected non-*Avr* control genes were used to investigate the genetic variations in 62 rice blast strains from different parts of China. Frequent presence/absence polymorphisms, high levels of nucleotide variation (~10-fold higher than non-*Avr* genes), high non-synonymous to synonymous substitution ratios, and frequent shared non-synonymous substitution were observed in the *Avr*-genes of these diversified blast strains. In addition, most *Avr*-genes are closely associated with diverse repeated sequences, which may partially explain the frequent presence/absence polymorphisms in *Avr*-genes.

**Conclusion:**

The frequent deletion and gain of *Avr*-genes and rapid non-synonymous variations might be the primary mechanisms underlying rapid adaptive evolution of pathogens toward virulence to their host plants, and these features can be used as the indicators for identifying additional *Avr*-genes. The high number of nucleotide polymorphisms among *Avr*-gene alleles could also be used to distinguish genetic groups among different strains.

## Background

Rice blast, caused by infection with the ascomycete fungus *Magnaporthe oryzae*, is one of the most devastating known rice diseases. It threatens the stability of rice production worldwide [1]. One of the most effective and economically viable ways of controlling this disease is the utilization of appropriate resistant cultivars that have major resistance genes against the rice blast [2]. So far, 85 blast *R* genes have been identified in rice, but the fungus *M. oryzae* is highly variable and often can overcome resistant cultivars within a few years of their initial deployment in the field [3]. The gene-for-gene hypothesis shows that a single plant *R*-gene product recognizes a unique avirulence protein [4]. A large number of studies have provided evidence that some Avr proteins can be recognized by plant R proteins. A strong and rapid immune response follows this recognition. This prevents further invasion [5-7]. In this way, it has been hypothesized that the ability to defeat rice *R*-genes may be due to the instability of *Avr-*genes in *M. oryzae*, including deletion and translocation of the genes, the insertion of transposons into the gene or promoter sequence, and point mutations [8-11].

Currently, 85 major rice blast genes have been genetically characterized and 19 of them have been cloned [6]. Frequent copy number variation, high levels of nucleotide diversity, and high non-synonymous to synonymous substitution ratios (high *Ka/Ks*, indicating positive selection) were observed at these *R*-gene loci, suggesting the rapid allelic diversification of these *R*-genes as an adaptive response to the rapidly changing spectrum of rice blast strains [12-14]. In rice blast pathogens, nine *Avr*-genes, all of which encode proteins of unknown function, have been cloned. These include *Avr-Pita*, *Avr-CO39*, *PWL1*, *PWL2*, *ACE1*, *Avr-Pizt*, *Avr-Pia*, *Avr-Pii*, and *Avr-Pik/km/kp*[5,9,10,15-17].

The direct interaction between *Pita* and *Avr-Pita* is the first binding effect observed between a plant R and a fungal Avr protein [18]. This *Avr*-gene is highly diversified and it has been confirmed that it is capable of rapid changes in nature, including complete or partial deletions, transposon insertions, point mutations, and even translocations [1]. In *PWL2*, a single nucleotide alteration leading to amino acid replacement has been shown to be crucial to its virulence: the protein of the deficient type *PWL2D* is more disordered and degenerative and cannot induce an immune response in the host plant [19]. *ACE1* is an *Avr* gene distinct from others in that it encodes an enzymatic protein that is involved in synthesis of a secondary metabolite and an allele with an insertion mutation that destroys its enzymatic function has also been found to deprive it of avirulence [9]. Among the recent cloned gene of *Avr-Pik/km/kp* showed frequent presence/absence polymorphisms and an excess of nonsynonymous substitutions [20]. Complete and effective alleles of *PWL1* and *Avr-CO39* are only present in Magnaporthe strains which cannot infect rice cultivars [8,16].

Because of their significant role in pathogen-host interactions, a total of four strains of *M. oryzae*, 70–15, Ina168, Y34, and P131, have been whole-genome sequenced or re-sequenced and made available to the public [20-22]. The first whole-genome sequenced strain 70–15 was generated by backcrossing progeny from a cross between a rice isolate and a weeping love grass isolate with the rice isolate Guy11 from France. The product of this backcrossing showed reduced female fertility and virulence [22]. EcoTilling experiments were performed to identify *Avr*-genes among the predicted secreted protein genes from the genome of 70–15. Results showed that only 22% of the 1032 pex genes contained nucleotide substitution in 46 diverse Oryza field isolates, but presence/absence polymorphisms might be more common in *Avr*-genes. For example, *Avr-Pia*, *Avr*-*Pii*, and *Avr*-*Pik/km/kp* are absent from the lab strain 70–15 and present in strain Ina168 [20]. The results of this study suggested that presence/absence polymorphisms might be the major mechanism underlying the evolution of rice blast *Avr*-genes. In this way, resequencing and association genetics might be a useful strategy for cloning additional *Avr*-genes [20].

Understanding the composition and dynamics of *Avr*-genes in natural populations from diverse rice-growing regions may be useful to breeders attempting to cultivate resistant varieties. Six well characterized *Avr-*genes and seven randomly selected control genes were used to investigate genetic variations in a single laboratory strain from Japan and 61 field strains from different parts of China.

## Results

### Presence/absence (P/A) polymorphisms in *Avr* genes

Thus far, no more than 10 *Avr* genes have been cloned from *M. oryzae*. In the present study, six well characterized *Avr* genes, including *Avr-Pita1*, *PWL2*,* Avr-Pik/km/kp*, *Avr-Pii*, *Avr-Pia*, and *ACE1*, and seven randomly selected non-*Avr* genes, which served as control genes (Table 1), were used to investigate the genetic variations in 62 rice blast strains, including 1 laboratory strain from Japan and 61 field strains from different parts of China (Additional file 1: Table S1). In each control gene, as expected, PCR-amplified products and their sequences were obtained from all of these 62 blast strains (Table 1). However, the PCR-amplified products and sequences were obtained in only some strains even after repeated efforts with different combinations of primers (Additional file 1: Table S2). This suggested that presence/absence polymorphisms might be prevalent at these *Avr* loci in these strains.

**Table 1:** Loci presence in blast strains from different regions at each locus

**Group**			**Strains carrying *Avr *** **genes**^**1**^						**Haplotype diversity**^**2**^
			**JS**	**YN**	**GD**	**SC**	**OTs**	**Total**	
	**Total numbers of strains**		**14**	**13**	**6**	**26**	**3**	**62**	
*AVR* genes	*AVR-Pita1 (881 bp)*		9	11	3	21	3	47	0.67
	*PWL2 (438 bp)*		7	10	6	21	2	46	0.46
	*AVR-Pik (342 bp)*		13	11	4	25	3	56	0.96
	*AVR-Pii (213 bp)*		1	0	1	8	0	10	0.16
	*AVR-Pia (276 bp)*		3	0	0	2	0	5	0
	*ACE1*^*3*^* (12694 bp)*	*KS* subunit	11	10	5	19	2	47	0.26
		*AT* subunit	12	7	4	19	1	43	0
Control genes	*CAL*		14	13	6	26	3	62	0
	*ACT*		14	13	6	26	3	62	0
	*ITS*		14	13	6	26	3	62	0.69
	*CHS1*		14	13	6	26	3	62	0.49
	*PTH11*		14	13	6	26	3	62	0
	*Hik*		14	13	6	26	3	62	0
	*ABC2*		14	13	6	26	3	62	0

^1^JS, Jiangsu province in east of China; YN, Yunnan province in southwest of China; GD, Guangdong province in south of China; SC, Sichuan province in midwest of China; OTs, three strains from other regions, one from Anhui province in east of China, one from Hubei province in central China and one from Japan, respectively.

^2^Haplotype diversity calculated by Nei 1987, equations 8.4 and 8.12 but replacing 2n by n.

^3^For *ACE1* gene, which is as large as 12 kb and can’t be cloned as a whole, we picked 2 subunits of the gene, β-ketoacyl synthase (*KS* subunit) and acyltransferase (*AT* subunit) as the tested subunits to imply the characteristics of the whole gene. From 37 strains have we successfully cloned both of the two loci.

*Avr-Pik/km/kp*, *Avr-Pii*, and *Avr-Pia* effector genes were first recognized by their *P/A* polymorphisms between laboratory strains of Ina168 and 70-15 [20]. In the present study, their alleles were found in 56, 10, and 5 out of the 62 strains, respectively (Table 1). This was consistent with previous reports that many *Avr* genes showed presence/absence polymorphisms in plant pathogens [20,23]. Notably, only 5 alleles were detected in *Avr-Pia* locus and 10 in *Avr-Pii* locus, even after another three primer pairs were used, including previously reported primers [23]. This suggested that *Avr-Pia* and *Avr-Pii* alleles were present in the sampled blast strains at a very low frequency. Although *P/A* polymorphisms have also been reported at the *Avr-Pik* locus, the highest frequency (56 out of 62) was observed in these sampled strains. The other three loci, *Avr-Pita1, PWL2* and *ACE1*, have moderate frequence of *P/A* polymorphisms in our sampled strains (Table 1). Overall, pattern of *P/A* polymorphisms at *Avr* loci showed a clear departure from the control genes in the present study.

### Frequency of nucleotide polymorphisms among *Avr* genes

Haplotype diversity can be used to measure the uniqueness of a particular haplotype in a given population. In the 7 control gene loci, no allelic diversity was detected in 5 gene loci among the 62 strains sampled (Table 1). However, diverse alleles were found in 5 of 6 *Avr* loci (haplotype diversity ranged from 0.16 to 0.96) except *Avr-Pia*, which is only present in 5 strains and has no diversity. The haplotype diversity reached 0.96 at the *Avr-Pik* locus, suggesting a high level of allelic diversity in this locus in the sampled strains.

A recent genome-wide study showed no nucleotide substitutions in ~82% (7569 out of 9184) of orthologous gene pairs among 3 whole-genome sequenced blast strains [21]. This suggested that most of these genes were well-conserved in different strains. The average levels of nucleotide diversity (π) were very low in those orthologous pairs (the average ranged from 0.00024 to 0.0006) between any pair of genomes. Similar results were also observed at the 7 control loci. Except fot ITS and CHS loci, no nucleotide substitution was detected at any of the 5 control loci so that only ITS and CHS loci are used in the diversity analysis; in which, the nucleotide diversity was 0.001 and 0.0012, respectively (Table 2). However, nucleotide substitutions were detected at all 5 *Avr* loci. The highest levels of nucleotide diversity (0.005) were observed at *Avr-Pita1* and *Avr-Pik*. Diversity was ~10-fold higher than the genome-wide average [22], suggesting that the *Avr* genes retain more genetic diversity than other genes. The most pronounced allelic diversity in *Avr* genes was observed in strains from Sichuan Province, which is in midwestern China, suggesting that strains from different regions have different levels of diversity even at the same *Avr* locus.

**Table 2:** **Nucleotide diversity of *Avr *** **and control genes**

	***Avr*-genes**									**Control loci**		
	***Avr-Pita1 (817 bp)***		***PWL2 903 bp***		***Avr-Pik 1371 bp***		***Avr-Pii 213 bp***	***ACE1 2193***		***CHS1 925 bp***	***ITS 771 bp***	**Others**
	**Non-coding 250 bp**	**CDS 567 bp**	**Non-coding 465**	**CDS 438 bp**	**Non-coding 1029 bp**	**CDS 342 bp**	**CDS 213 bp**	**Non-coding 87 bp**	**CDS 2106 bp**	**CDS 925 bp**	**All region**	**All region**
JS	0	0.002	0	0.001	0.001	0.004	/	0	0	0	0.0008	0
GD	0	0.001	0.002	0	0.001	0.003	/	0.0035	0.003	0.001	0.0005	0
SC	0.002	0.006	0.001	0.001	0.001	0.005	0.0003	0.0021	0.002	0.001	0.0012	0
YN	0	0	0.001	0	0	0.004	/	0	0	0	0.0002	0
**All sequences**	0.002	**0.005**	0.001	0.001	0.001	**0.005**	0.0003	0.001	0.001	0.001	0.0012	0

ITS isn’t a protein coding gene and doesn’t have a CDS sequence. The bold style reflects that the diversity of CDS region is significantly larger than that of non-coding region.

No frame-shift or premature stop mutations were found in any of the sampled *Avr* or control loci. This is in contrast to previous reports, in which frequent frame-shift and premature stop mutations were found in pathogen *Avr* genes and plant resistance genes [1,23,24]. No frequent insertion/deletion polymorphisms were observed in these strains. Only one deletion polymorphism was detected in the first exon region of *Avr-Pita1* allele in strain SC3. It was three nucleotides in length. In addition, significantly more nucleotide substitutions were found in coding-sequence regions than in non-coding sequence regions at *Avr-Pita1* and *Avr-Pik* loci (Table 2), suggesting that diversifying selection took place in the CDS regions of these two *Avr* loci. This is consistent with a recent study, in which different groups of strains are collected worldwide and the diversity of coding region is larger than that of non-coding region in each group [25].

However, no significant difference was detected between nucleotide diversity within regions and divergence of strains between different regions, except for *Avr-Pita1* locus (Additional file 1: Table S3). In *Avr-Pita1* alleles, significantly higher levels of *D*_*xy*_ were observed than nucleotide diversity within regions. This was especially true of combinations of GD with other regions, suggesting more pronounced differentiation of blast strains between GD and other regions at the *Avr-Pita1* locus.

### Non-synonymous (Ka) and synonymous (Ks) nucleotide substitutions at *Avr* loci

In one recent study, a total of 703 gene loci (7.6%) were detected with *Ka > Ks* from 9184 orthologous gene pairs among three whole-genome sequenced blast strains. These included 6 gene pairs for which *Ka/Ks* > 1 and 697 gene pairs with only non-synonymous substitutions^19^. However, *Ka* was found to be greater than *Ks* for 4 of the 5 *Avr* loci sampled here (Table 3). This suggested that these *Avr* alleles had undergone strong positive selection. Especially among *Avr-Pik* alleles, 7 non-synonymous substitutions and no synonymous substitution were found in strains from any region (Table 3). This suggested a strong positive selection at this locus. At the *Avr-Pita1* locus, *Ka* was found to be greater than *Ks* in strains from three regions, GD, JS, and SC, and no nucleotide substitutions were detected in strains from YN. Higher levels of nucleotide diversity were also observed at *Avr-Pita* and *Avr-Pik* loci, suggesting that the strong positive selection might drive the rapid diversification of *Avr* genes.

**Table 3:** **Non-synonymous (*Ka*) and synonymous (*Ks*) nucleotide substitutions at *Avr *** **and control loci**

**Regions**	***Avr-Pita1***			***PWL2***			***Avr-Pik***			***ACE1-KS***			***CHS***		
	***Ks***	***Ka***	***Ka/Ks***	***Ks***	***Ka***	***Ka/Ks***	***Ks***	***Ka***	***Ka/Ks***	***Ks***	***Ka***	***Ka/Ks***	***Ks***	***Ka***	***Ka/Ks***
JS	0	0.002	*Ka > Ks*	0	0.002	*Ka > Ks*	0	0.005	*Ka > Ks*	0	0	–	0	0	–
GD	0	0.002	*Ka > Ks*	0	0	–	0	0.004	*Ka > Ks*	0.012	0	0	0	0.001	*Ka > Ks*
SC	0.004	0.007	1.75	0	0.001	*Ka > Ks*	0	0.006	*Ka > Ks*	0.007	0	0	0	0.002	*Ka > Ks*
YN	0	0	–	0	0	–	0	0.005	*Ka > Ks*	0	0	–	0	0	–
**All sequences**	0.003	0.006	2	0	0.001	*Ka > Ks*	0	0.006	*Ka > Ks*	0.004	0	0	0	0.001	*Ka > Ks*

*Avr-Pii* only has 10 sequences and it’s not informative enough to calculate Ks and Ka at this locus. So this locus is removed from the nonsynonymous/synonymous analysis.

”-“ means Ks and Ka both equal to zero or Ka/Ks is not able to be calculated.

### Genetic exchanges indicated by phylogenetic trees of *Avr* alleles

**Figure 1:**
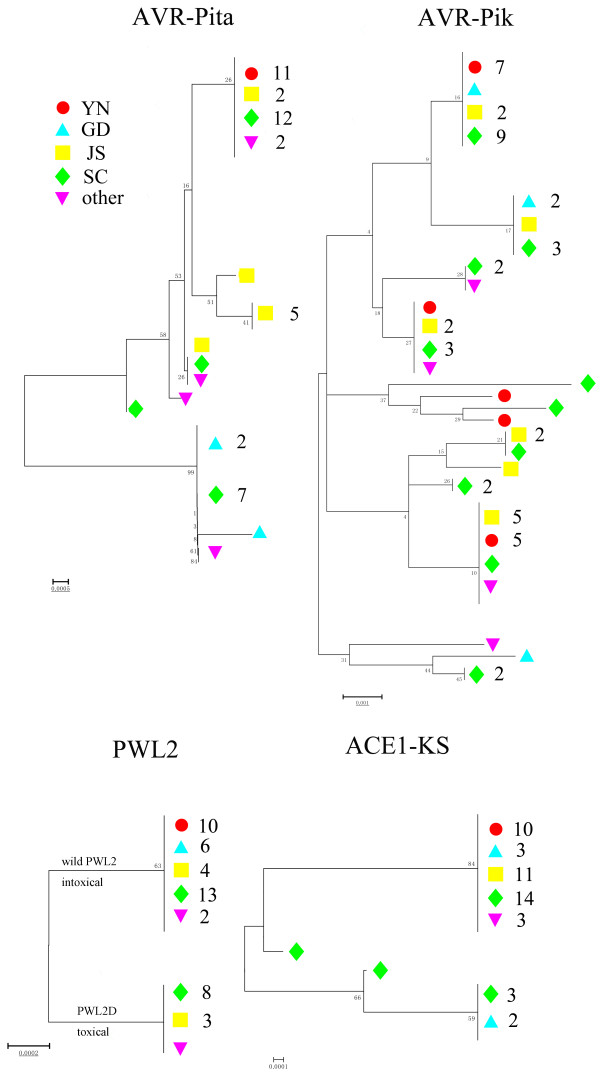
**The phylogenic trees of *Avr*-genes.** In this Figure, each tree represents the phylogenetic relationship of one *AVR* gene. The colored shape indicates the origin of the strain, and the number of strains is labeled behind the shape if there are more than one member at the the same position. In the tree of *PWL2*, there are only two kinds of alleles. One of them is the wild type, which is intoxical, while the other is inefficient and toxical.

To further clarify the phylogenetic relationships among *Avr* alleles, neighbor-joining trees were constructed based on nucleotide variations at each locus. These trees revealed substantial variations from tree shapes and depth, indicating a wide range of evolutionary histories. We performed this analysis for *Avr-Pita*, *PWL2*, *Avr-Pik* and *ACE1*, but not for *Avr-Pia* and *Avr-Pii* due to their small samples (only 5 and 10 strains). The *Avr-Pik* and *Avr-Pita1* loci were found to possess many low-frequency variants, but only a small number of them contained multiple alleles at intermediate frequencies (Figure 1). There was also a higher level of non-synonymous substitutions at these two *Avr* loci. This might indicate that positive selection acted on these two *Avr* genes.

At the *PWL2* and *ACE1* loci, two major phylogenetic subclades were observed in each tree, suggesting that a large number of them contained multiple alleles at intermediate frequencies. Only one non-synonymous nucleotide polymorphism was found between these two subclades at the *PWL2* locus (Figure 1), and this non-synonymous variation has been proven to be a key substitution for gene avirulence and virulence that results in recognition or loss of recognition of the pathogen by the host plants [17]. In other words, three types of groups, absence, toxic, and non-toxic, were found in all of blast strains at this locus, and the toxic and non-toxic groups were clustered in two distinct clades, suggesting that these virulence and avirulence functional alleles might be maintained by balancing selection. Similar results were observed in *ACE1* locus.

These *Avr*-gene phylogenetic trees showed another relevant characteristic: theoretically, the distribution of blast strains should show significant clustering with the geographic structure of these strains. However, strains from the same region did not cluster in the same clade (Figure 1). Rather, they were scattered among many mixed clades, indicating that shared polymorphisms and haplotypes were widespread among different regional strains. To confirm this, 26 additional *Avr-Pita1* amino acid sequences collected from different countries were retrieved from GenBank and used to construct a NJ tree with the blast sequences from the present study [1]. The topology was found to be consistent with the phylogenic tree of sequences from the present strains (Figure 1 and Additional file 1: Figure S1). The additional haplotypes from the U.S., Colombia, Egypt, India, and the Philippines were also scattered across the phylogenic tree, suggesting that frequent genetic exchanges across different parts of China and even different continents may be widespread at these *Avr-*gene loci. Interestingly,a recent study [25] has shown that strains from Thailand tend to cluster in the phylogenic tree at *AVR-Pita1* locus, suggesting different evolutionary pattern or selective pressure at this locus in the strains of Thailand.

Because the genome-wide genetic diversity is very slight between blast strains it is difficult to distinguish the population structure using these limited markers [21]. The high number of nucleotide polymorphisms among *Avr* gene alleles could be used to distinguish genetic groups across different strains by using the sequences from single or multiple *Avr*-gene loci. A phylogenetic tree showing 62 rice blast strains was constructed by nucleotide sequences from these 4 *Avr* loci. In this tree, each blast strain was distinguished by *Avr*-allelic sequences, and these sequences could be used to identify different blast strains.

## Discussion

### Contribution of presence/absence polymorphisms in *Avr*-genes to the rapid adaptive evolution of rice blast pathogens

To date, more than 80 rice blast resistance genes have been identified in various rice cultivars [26]. However, many resistance genes lost their effectiveness within a short period of time because of the high levels of variability among the *Avr* genes in the blast strains, suggesting rapid adaptation by the pathogen [18,27-29]. Previous studies have shown that *Avr*-genes might be undergoing frequent mutational events, including spontaneous deletions, nucleotide substitutions, and inactivation by TE insertions, which can lead to loss of avirulence [1,27]. A genome-wide survey showed that about half of the characterized blast *Avr*-genes, including *Avr-Pik*, *Avr-Pii*, and *Avr-Pia*, were absent in the assembled blast 70–15 genome, and the presence/absence polymorphisms might be the major mechanisms in evolution of *Avr*-genes [20].

Despite repeated efforts with different primer combinations, the amplification of the six *Avr*-genes evaluated in this study failed in many strains (average on 41% and ranging from 9.7% at *Avr-Pik* locus to 92% at *Avr-Pia* locus), but the PCR-amplified products and sequences of all control genes were obtained from all 62 blast strains (Table 1). This suggested that presence/absence polymorphisms might be prevalent at *Avr* loci in these sampled strains. Previous studies have shown that *Avr-Pita*, *Avr-Pia*, *Avr-Pik*, and *Avr-Pii* are highly variable in their genome location and have undergone multiple translocations in their genomes [29].

**Figure 2:**
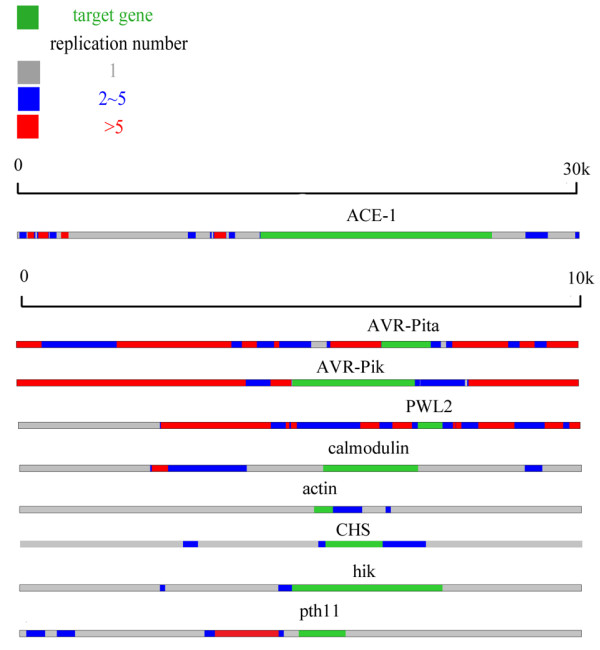
**The genomic composition of the flanking sequences.** The copy numbers of the flanking sequences of different loci are shown in this Figure. Each line displays the 10 kb flanking sequence of a gene (for *ACE1* the flanking length is 30 kb as this gene is too long). The green region shows the position of the target gene. the red region indicates that this part of sequence has more than 5 copies in the whole genome while grey region only has one copy. Blue region has copy numbers ranging from 2 to 5.

In order to explain the prevalence of presence/absence polymorphisms and multiple translocations across the genome in these *Avr*-genes, the genomic composition of the flanking sequences was surveyed in the area around 4 *Avr*-genes and 5 control genes. As expected, a large number of repetitive sequences with at least 5 copies were found in the flanking sequences of 3 *Avr*-genes, specifically *Avr-Pita*, *Avr-Pik*, and *PWL2* (Figure 2), suggesting that most *Avr*-genes are closely associated with diverse repeated sequences, including transposable elements. However, the flanking sequences surrounding the 5 randomly selected non-*Avr* genes (control genes) were non-repetitive (Figure 2). This may partially explain why frequent presence/absence polymorphisms and translocations across the genome are detected in *Avr*-genes. These results also indicated that the frequent deletion of avirulence genes (presence/absence polymorphisms) might be the primary mechanism underlying the rapid adaptation of pathogens toward virulence to their host plants. The translocation of *Avr*-genes may be associated with frequent recovery through transfer from other individuals, suggesting that the pronounced mobility of *Avr* might be the key mechanism underlying the rapid adaptation toward plant *R*-genes [29].

### Levels of non-synonymous substitutions and positive selection

To identify *Avr*-genes, a total of 1032 of predicted secreted protein genes in strain of 70–15 were tested using EcoTilling experiments in 46 diverse *Oryza* field isolates. Approximately 78% of them showed no nucleotide polymorphisms [20]. A similar result was observed in another study, in which no nucleotide substitutions were detected in ~82% (7569 out of 9184) of orthologous gene pairs among three whole-genome sequenced blast strains [21]. However, among the 6 well-characterized *Avr-*genes evaluated in this study, nucleotide substitutions were detected in 5 out of 6 *Avr* loci. In *Avr-Pita1* and *Avr-Pik*, the nucleotide diversity (0.005) was ~10-fold more pronounced than the genome-wide average, suggesting that the *Avr*-genes retain considerably more genetic diversity than other genes in this genome.

In the 9184 orthologous gene pairs, *Ka* was found to be greater than *Ks* for only 703 gene loci (~7.6%). This included 6 pairs of genes for which *Ka/Ks* > 1 and 697 pairs with only non-synonymous substitutions [21]. However, among the *Avr*-genes sampled here, *Avr-Pita1*, *Avr-Pik*, and *PWL2* showed an excess of non-synonymous substitutions for which *Ka > Ks* (Table 3). This suggested that these *Avr* alleles had undergone strong positive selection.

At the *Avr-Pita1* and *Avr-Pik* loci, high levels of nucleotide substitutions and presence/absence polymorphisms were detected, and most variations in the DNA sequence were observed in the exon regions, most of them in the form of amino acid substitutions (Table 3). Only 7 nonsynonymous polymorphic sites were found in *Avr-Pik* alleles, suggesting that these *Avr-*genes are under positive selection and that the rapid variations in these alleles might be responsible for defeating race-specific resistance.

In one recent study, 5 *Avr-Pik* alleles were observed. They had 5 nonsynonymous polymorphic sites in 21 Japanese blast isolates [30]. The three major alleles, D, E, and A, are likely to have different levels of toxity and arose at different time. A is likely to be newly derived. Strain A often escapes from the surveillance of *Pikp* and Pik*, given that the ancestral type D fails to overcome the *Pik* alleles [30]. The authors propose that the *Avr-pik* and *Pik* are locked into an arms race driven by co-evolution. And these are also the three major alleles in our result. What’s more, we also find some other alleles.

Two additional amino acid replacements, 2-H and 103-G (marked in blue in Additional file 1: Figure S2) were found in these strains. The replacement of arginine by histidine at the second position was found to occur within the signal peptide region. This region is highly significant to the secretion of *Avr-Pik*. This replacement may influence the interactions between *Avr*- and *R*-proteins in the plant hosts. This is consistent with the results of a previous study that showed that OT2, a strain containing this replacement, showed stronger pathogenicity than the strains of *Avr-Pik-A*. Some new combinations of amino acid alleles were also observed, indicating pronounced diversity among protein sequences. The rapid evolution of new strains might be attributed to rapid loss of function of avirulence effector genes that correspond to the resistance genes in a gene-for-gene manner [4,29].

At the *PWL2* locus, only one non-synonymous substitution was detected. It has been proven to be a key substitution for avirulence or virulence function. Interestingly, two candidate *R*-genes might be responsible for the recognition of the *Avr*-gene of *PWL2* (Additional file 1: Table S4). These genes are resistant to most of the strains with the *PWL2* genotype and are susceptible to the strains with the *PWL2D* genotype.

### Estimation of the population genetics of blast strains

Though high-throughput sequencing is a highly valuable form of DNA sequencing, genome-wide genetic diversity between rice blast strains is so slight that the cost of using high-throughput sequencing to determine population structure becomes prohibitive [20,21]. The data collected in the current work showed that the high level of nucleotide polymorphisms among *Avr*-gene alleles could be used to distinguish genetic groups among different strains by using the sequences from single *Avr*-gene loci or multiple *Avr*-gene loci, and each blast strain was distinguished using *Avr*- allelic sequences at the 5 of 6 *Avr* loci (Additional file 1: Figure S3).

## Conclusions

In the study of 6 *Avr*-genes in 62 *Magnaporthe oryzae* strains, a prevalence of presence/absence polymorphisms is observed, which may be crucial for the infestants to escape from the immune systems of the hosts. This may partly be explained by the fact that most *Avr*-genes are closely associated with diverse repeated sequences. Relatively high rate of nonsynonymous replacements are discovered and some of the polymorphisms have been proved responsible for the alteration of the gene function. The frequent present/absent polymorphisms and SNP of *Avr*-genes could be used to distinguish genetic groups between different strains.

## Methods

### Strains of *Magnaporthe* oryzae

A total of 62 different *M. oryzae* strains from different parts of China were collected and used in *Avr-*gene amplification (Additional file 1: Table S1). These strains represent most of the major areas of rice production in China reasonably well.

### DNA extraction and PCR amplification

Each strain of *M. oryzae* was first cultured on PDA medium in tubes and then transferred to PDB medium in conical flasks to harvest hyphae. They were kept in a shaking incubator at a speed of 180 rpm at 26°C for 48 h, after which the flasks were full of balls of hyphae. These were removed by filtration and then baked out with vacuum dryer at 30°C. The dried hyphae were ground to powder in mortar with liquid nitrogen. The powder was transferred to microtubes and processed with a DNeasy Plant Mini Kit from Qiagen to produce the DNA template.

To determine the sequence of these loci, a standard PCR process was carried out as follows: Primers were designed to make products 500–1000 bp in length that included the target gene of the coding sequences. When sequencing failed, more primers were brought out and different conditions were tried. All primers are listed in Additional file 1: Table S2. The reaction was carried out in a 20 μl LA Taq system containing 2.6 μl of template, 3.2 μl of dNTP (2.5 mM each, Takara), 10 μl of GC buffer (2X, Takara), 0.2 μl of LA Taq (5 μ/μL, Takara), 2 μl of upstream and downstream primers at concentration of 2 μmol/L. PCR products were sequenced on an ABI3100A automated sequencer.

### Data analysis

The amino acid sequences were first aligned using the MUSCLE software package with default options [31]. The resulting amino acid sequence alignments were then used to guide the alignments of the corresponding nucleotide coding sequences. Based on the nucleotide alignment results, phylogenetic trees were constructed using the bootstrap neighbor-joining method with Kimura two-parameter model in MEGA [32]. The stability of internal nodes was assessed by bootstrap analysis with 1,000 replicates. Haplotype diversity, nucleotide diversity with the Jukes and Cantor correction, and nonsynonymous and synonymous substitutions were calculated with DnaSP [33]. To determine the number of replications of the region adjacent to target loci, a 10 kb sequence containing the target locus from the 70–15 genome was selected (a 30 kb sequence was selected for ACE1) and used to find all copies of each fragment in any part of the blast genome.

## Competing interests

The authors declare that they have no competing interests.

## Authors’ contributions

SY, PL and JH designed the study. JH and WS contributed extensively to the bioinformatics analyses. WS and QD performed PCR experiments. SY, WS and JH wrote the manuscript. SY, JH and PL prepared and revised the manuscript. All authors read and approved the final manuscript.

## Supplementary Material

Additional file 1: Table S1 *M.grisea* strains information. **Table S2.** Primers used in this study. **Table S3.** Dxy values of loci of different pairs of regions. **Table S4.** Filtering results based on PWL2 allele types. **Figure S1.** The complementary phylogenetic tree of AVR-Pita. This tree contains the AVR-Pita sequences of our study and 26 amino acid sequences from Genbank. The additional sequences are tagged with the country which have strains carrying this allele. **Figure S2.** The complementary phylogenic tree of *Avr-Pik*. This tree contains sequences retained in our study and 5 of 6 sequences reported in the previous study. There are 7 SNPs among these alleles (table on the left). 5 of them (yellow) have been reported before and 2 of them (blue) are discovered in our study. For some representative sequences, the SNPs they have are shown in the table on the right. **Figure S3.** The phylogenic tree consists of 4 *AVR* genes. This tree is constructed by combination of *AVR-Pita, PWL2, AVR-Pik* and *ACE1 AVR-Pia* and *AVR-Pii* have too many sequences so that are excluded. Click here for file
