# Supplementary material for: Rapid evolution of avirulence genes in rice blast fungus Magnaporthe oryzae
Source: BMC Genet. 2014 Apr 11;15:45. doi: 10.1186/1471-2156-15-45 (PMC4021558; doi:10.1186/1471-2156-15-45)

Additional file 1: Table S1 *M.grisea* strains information

| strain ID | Region code | | origin |
| --- | --- | --- | --- |
| JS2 | JS | | Jiangsu province,east of China |
| JS3 | JS | | Jiangsu province,east of China |
| JS5 | JS | | Jiangsu province,east of China |
| JS11 | JS | | Jiangsu province,east of China |
| JS15 | JS | | Jiangsu province,east of China |
| JS16 | JS | | Jiangsu province,east of China |
| JS17 | JS | | Jiangsu province,east of China |
| JS19 | JS | | Jiangsu province,east of China |
| JS20 | JS | | Jiangsu province,east of China |
| JS21 | JS | | Jiangsu province,east of China |
| JS22 | JS | | Jiangsu province,east of China |
| JS31 | JS | | Jiangsu province,east of China |
| JS32 | JS | | Jiangsu province,east of China |
| JS33 | JS | | Jiangsu province,east of China |
| GD1 | GD | | Guangdong Province,south of China |
| GD2 | GD | | Guangdong Province,south of China |
| GD3 | GD | | Guangdong Province,south of China |
| GD4 | GD | | Guangdong Province,south of China |
| GD5 | GD | | Guangdong Province,south of China |
| GD6 | GD | | Guangdong Province,south of China |
| SC1 | SC | | Sichuan Province,midwest of China |
| SC2 | SC | | Sichuan Province,midwest of China |
| SC3 | SC | | Sichuan Province,midwest of China |
| SC4 | SC | | Sichuan Province,midwest of China |
| SC7 | SC | | Sichuan Province,midwest of China |
| SC8 | SC | | Sichuan Province,midwest of China |
| SC9 | SC | | Sichuan Province,midwest of China |
| SC10 | SC | | Sichuan Province,midwest of China |
| SC13 | SC | | Sichuan Province,midwest of China |
| SC15 | SC | | Sichuan Province,midwest of China |
| SC16 | SC | | Sichuan Province,midwest of China |
| SC17 | SC | | Sichuan Province,midwest of China |
| SC18 | SC | | Sichuan Province,midwest of China |
| SC19 | SC | | Sichuan Province,midwest of China |
| SC20 | SC | | Sichuan Province,midwest of China |
| SC21 | SC | | Sichuan Province,midwest of China |
| SC22 | SC | | Sichuan Province,midwest of China |
| SC23 | SC | | Sichuan Province,midwest of China |
| SC24 | SC | | Sichuan Province,midwest of China |
| SC25 | SC | | Sichuan Province,midwest of China |
| SC26 | SC | | Sichuan Province,midwest of China |
| SC27 | SC | | Sichuan Province,midwest of China |
| SC28 | SC | | Sichuan Province,midwest of China |
| SC29 | SC | | Sichuan Province,midwest of China |
| SC30 | SC | | Sichuan Province,midwest of China |
| SC31 | SC | | Sichuan Province,midwest of China |
| OT1 | OT | | Japan,a lab strain used in resistance tests |
| OT2 | OT | | Hubei Province,Central China |
| OT3 | OT | | Anhui Province,east of China |
| Y11 | YN | Yunnan province,southwest of China | |
| Y12 | YN | Yunnan province,southwest of China | |
| Y13 | YN | Yunnan province,southwest of China | |
| Y14 | YN | Yunnan province,southwest of China | |
| Y1 | YN | Yunnan province,southwest of China | |
| Y2 | YN | Yunnan province,southwest of China | |
| Y3 | YN | Yunnan province,southwest of China | |
| Y4 | YN | Yunnan province,southwest of China | |
| Y6 | YN | Yunnan province,southwest of China | |
| Y7 | YN | Yunnan province,southwest of China | |
| Y8 | YN | Yunnan province,southwest of China | |
| Y9 | YN | Yunnan province,southwest of China | |
| Y10 | YN | Yunnan province,southwest of China | |

Additional file 1: Table S2 Primers used in this study

| group | locus | primers | Sequence(5’-3’) |
| --- | --- | --- | --- |
| Control  genes | *ABC2* | U1 | 5’TTG AGA AAT ATC GCG GAG AG-3’ |
|  |  | D1 | 5’GCG GAA CAA GTT GGT CAT AA-3’ |
|  |  | *U2 | 5’GCT GCG TGT ACT TTG GTG AC-3’ |
|  |  | *D2 | 5’AGT GGC GTG TAG AAT TGG TC-3’ |
|  | *CHS* | *U1 | 5’TCA CCC ATA TGC GCT ACA CT-3’ |
|  |  | *D1 | 5’CAG GCA CAT CAG TTT CTC CA-3’ |
|  | *ACT* | U | 5’ATGTGCAAGGCCGGTTTCGC-3’ |
|  |  | D | 5’TACGAGTCCTTCTGGCCCAT-3’ |
|  | *CAL* | U | 5’GAGTTCAAGGAGGCCTTCTCCC-3’ |
|  |  | D | 5’CATCTTTCTGGCCATCATGG-3’ |
|  | *PTH11* | U | 5’AAG AGT TCT GCG CCA TCA TC-3’ |
|  |  | D | 5’GAA TTA CGT GCA CTG CCC AT-3’ |
|  | *Hik* | U1 | 5’CCA TAA CTA CCA CCG CAG AT-3’ |
|  |  | D1 | 5’GAT CTT TCG GCT CAT GTC AC-3’ |
|  |  | *U2 | 5’GGA GAT TAC CAC CGA TGT CA-3’ |
|  |  | *D2 | 5’GCC TCG ATA CAC TTC TCA CG-3’ |
| AVR  Genes | *AVR_Pita* | *U1 | 5’GAC CCG TTT CCG CCT TTA TT-3’ |
|  |  | *D1 | 5’GAT TCC CTC CAT TCC AAC AC-3’ |
|  |  | U2 | 5’CGA CCC GTT TCC GCC-3’ |
|  |  | D2 | 5’ACC CTA ACC CTA ACC CTC TAT TGT TAG ATT GAC C-3’ |
|  |  | U3 | 5’GCG ATT TCG GCC TTC ACC-3’ |
|  |  | D3 | 5’TGA CCG CGA TTC CCT CCA TT-3’ |
|  | *AVR_PWL2* | *U1 | 5’CTC CGC CAC TTT TCT CAT TC-3’ |
|  |  | *D1 | 5’GCC CTC TTC TCG CTG TTC AC-3’ |
|  |  | U2 | 5’CGGTGGCGGGTGGACTAACA-3’ |
|  |  | D2 | 5’CCGCCTCCGTGATTTCCGTA-3’ |
|  | *AVR_Pik* | 1421U | 5’GTC AAT ATG CGT GTT ACC AC-3’ |
|  |  | *1812L | 5’GTC AAC CAA GCG TAA ACC TC-3’ |
|  |  | *701U | 5’CGA TTC AGA AGT TAG GCA TT-3’ |
|  |  | U2 | 5’TTGAAGAGGGGGCGGATAA-3’ |
|  |  | D2 | 5’GCATTGACGACAGCGACAGT-3’ |
|  | *AVR_pii* | *208U | 5’GAG GCC GAT ATG TTA CGA TT-3’ |
|  |  | *1187L | 5’CTC TGC TCT CAC GCT TTA CC-3’ |
|  |  | U2 | 5’TTTGGTAGATATCCGCTGAC-3’ |
|  |  | D2 | 5’CCGCAATAGTCCATAACTG-3’ |
|  | *AVR_Pia* | *96U | 5’GCC GCT AGC TGT ATA GAC AA-3’ |
|  |  | 157U | 5’CGC TTG AGA TTC TTT GCT AG-3’ |
|  |  | *1069D | 5’TCA TCG TCG AGT GGT GTA GG-3’ |
|  |  | U2 | 5’CGCCAGCCCCATACAC-3’ |
|  |  | D2 | 5’AGCTGCTGCTCTTCCGTTTT-3’ |
|  | *ACE1-at* | *U1 | 5’GAG GTG CCA GAT ATG TCG TC-3’ |
|  |  | *D1 | 5’GGA TGA GCA GAT GAG CAA CA-3’ |
|  |  | U2 | 5’GCCAACATCTCACAGACCGT-3’ |
|  |  | D2 | 5’CGGCAGTCCAGATTGGTCAT-3’ |
|  | *ACE1-Ks* | *U1 | 5’GCA CCT TGA CGT TTG AAC AG-3’ |
|  |  | *D1 | 5’TGA GTT TGC ATT GAG CGA GT-3’ |
|  |  | U2 | 5’GCGGCTGGAATCACC-3’ |
|  |  | D2 | 5’TCAAGAAAATTAGCCAACCC-3’ |

When have trouble in sequence cloning, we designed more primers and the primer pairs that produce the sequence that were finally used in sequence analysis hadbeen tagged with “*”.

| gene    pair of  regions | | AVR-Pita | PWL2 | AVR-Pik | ACE1-KS | CHS | ITS |
| --- | --- | --- | --- | --- | --- | --- | --- |
| SC | JS | 0.0060 | 0.0011 | 0.0050 | 0.0010 | 0.0010 | 0.00113 |
| SC | GD | 0.0090 | 0.0009 | 0.0040 | 0.0020 | 0.0000 | 0.00123 |
| SC | YN | 0.0040 | 0.0009 | 0.0050 | 0.0010 | 0.0010 | 0.00117 |
| JS | GD | 0.0130 | 0.0010 | 0.0060 | 0.0020 | 0.0010 | 0.00177 |
| JS | YN | 0.0020 | 0.0010 | 0.0040 | 0.0000 | 0.0020 | 0.00172 |
| GD | YN | 0.0130 | 0.0000 | 0.0070 | 0.0020 | 0.0010 | 0.00030 |
| p-value of the difference | | *0.026 | 0.243 | 0.072 | 0.425 | 0.145 | 0.165 |
| Dxy(average) | | 0.007833 | 0.000817 | 0.005167 | 0.001333 | 0.001 | 0.00122 |
| Pi value (all) | | 0.005 | 0.001 | 0.005 | 0.001 | 0.001 | 0.0012 |

Additional file 1: Table.S3 Dxy values of loci of different pairs of regions

Dxy values of most of the loci between different regions are still larger than that between 70-15 and Ina168. Moreover, AVR-Pita has Dxy values that are significantly larger than the Pi value within region, which is not observed in Pwl2, KS and CHS because Dxy values of other loci are close to Pi values.

Additional file 1: Table S4. Filtering results based on PWL2 allele types

| Loci | Donor | Receptor | *PWL2D* clade (virulent) | *PWL2* clade (avirulent) | | |
| --- | --- | --- | --- | --- | --- | --- |
|  |  |  | OT1 | Y11 | JS19 | JS21 |
| Os04g30930 | Tetep | TP309 | *S* | *R* | *R* | *R* |
| Os03g37720 | Gumei 2 | TP309 | *S* | *R* | *R* | *R* |

*R*, Resistance; *S*, Susceptibility. Phenotypes are based on the unpublished observations in our previous screen of rice blast isolates.

**Additional file 1: Figure S1.** The complementary phylogenetic tree of AVR-Pita.

This tree contains the AVR-Pita sequences of our study and 26 amino acid sequences from Genbank. The additional sequences are tagged with the country which have strains carrying this allele. ().


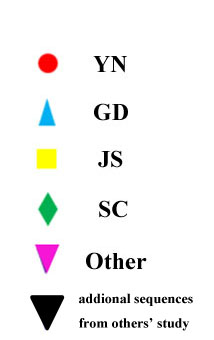

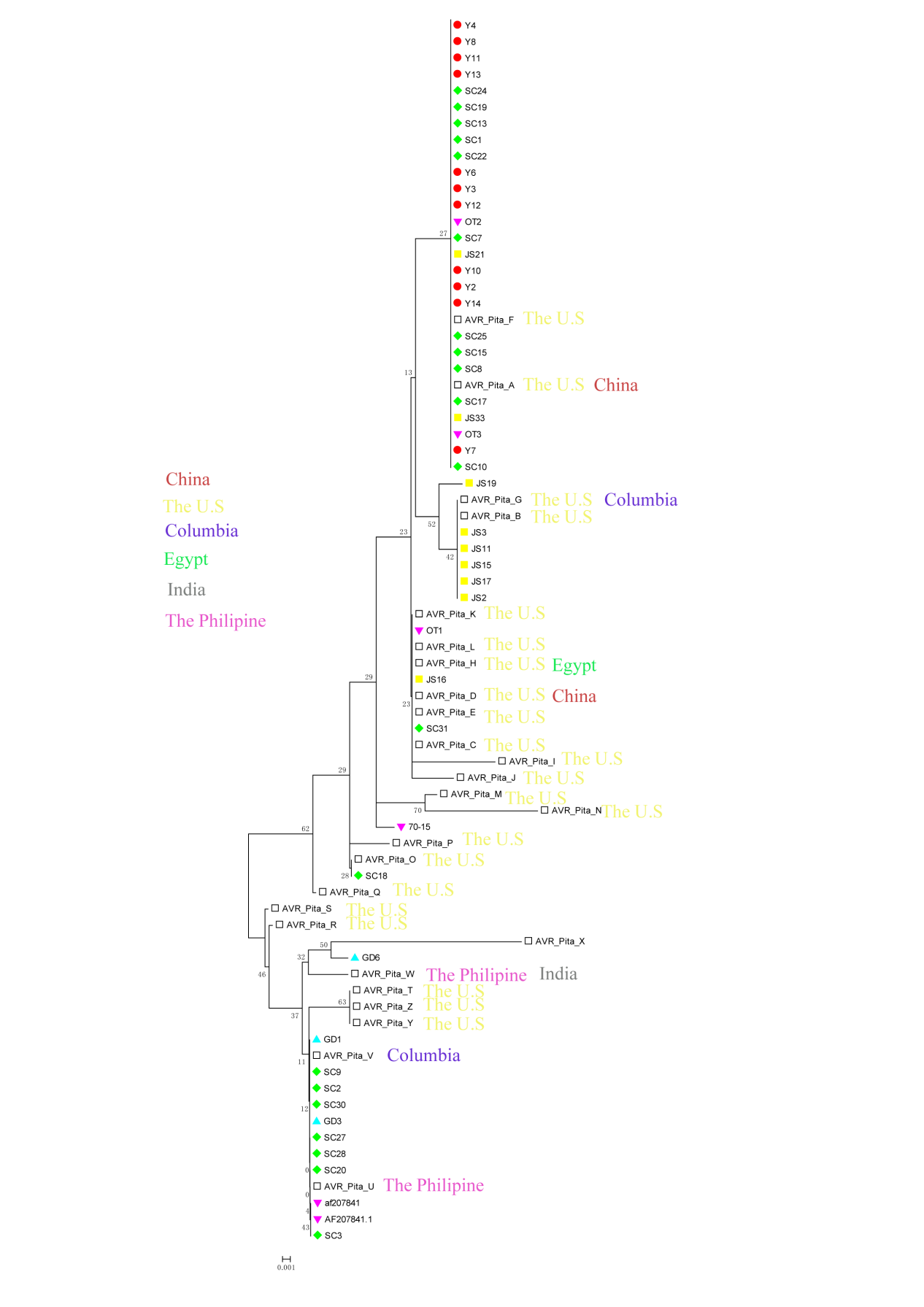


**Additional file 1: Figure S2. The** complementary phylogenic tree of *Avr-Pik* This tree contains sequences retained in our study and 5 of 6 sequences reported in the previous study. There are 7 SNPs among these alleles(table on the left). 5 of them(yellow) have been reported before and 2 of them(blue) are discovered in our study.

For some representative sequences, the SNPs they have are shown in the table on the right..

Additional file 1: Figure.S3 The phylogenic tree consists of 4 *AVR* genes.

This tree is constructed by combination of *AVR-Pita,PWL2,AVR-Pik* and *ACE1*

*AVR-Pia* and *AVR-Pii* have too many sequences so that are excluded.


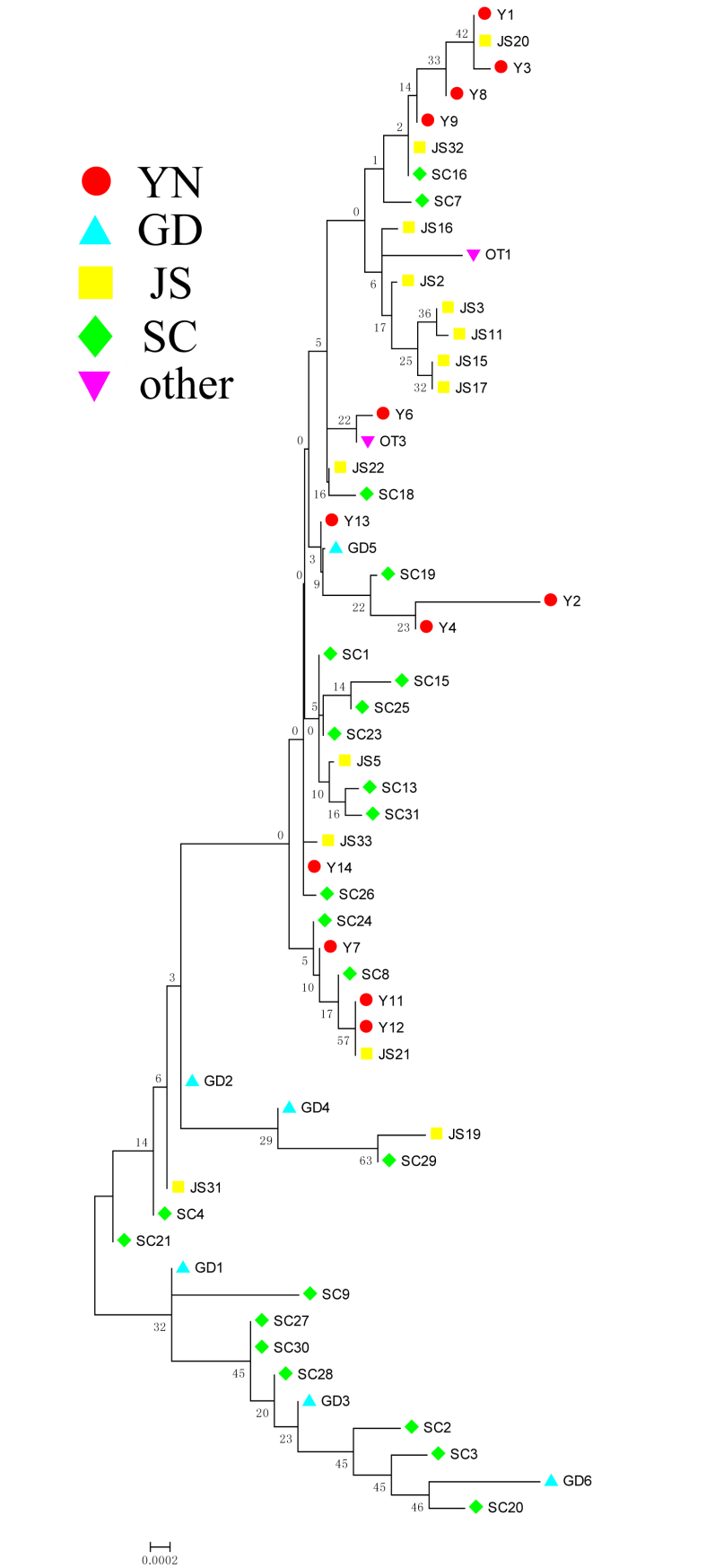

Supplement: Additional file 1: Table S1 — M.grisea strains information. Table S2. Primers used in this study. Table S3. Dxy values of loci of different pairs of regions. Table S4. Filtering results based on PWL2 allele types. Figure S1. The complementary phylogenetic tree of AVR-Pita. This tree contains the AVR-Pita sequences of our study and 26 amino acid sequences from Genbank. The additional sequences are tagged with the country which have strains carrying this allele. Figure S2. The complementary phylogenic tree of Avr-Pik. This tree contains sequences retained in our study and 5 of 6 sequences reported in the previous study. There are 7 SNPs among these alleles (table on the left). 5 of them (yellow) have been reported before and 2 of them (blue) are discovered in our study. For some representative sequences, the SNPs they have are shown in the table on the right. Figure S3. The phylogenic tree consists of 4 AVR genes. This tree is constructed by combination of AVR-Pita, PWL2, AVR-Pik and ACE1 AVR-Pia and AVR-Pii have too many sequences so that are excluded. [file 1471-2156-15-45-S1.docx]
